# Novel, Deep-Branching Heterotrophic Bacterial Populations Recovered from Thermal Spring Metagenomes

**DOI:** 10.3389/fmicb.2016.00304

**Published:** 2016-03-15

**Authors:** Daniel R. Colman, Zackary J. Jay, William P. Inskeep, Ryan deM. Jennings, Kendra R. Maas, Douglas B. Rusch, Cristina D. Takacs-Vesbach

**Affiliations:** ^1^Department of Biology, University of New MexicoAlbuquerque, NM, USA; ^2^Thermal Biology Institute and Department of Land Resources and Environmental Sciences, Montana State UniversityBozeman, MT, USA; ^3^Center for Genomics and Bioinformatics, Indiana UniversityBloomington, IN, USA

**Keywords:** Aquificales, hot springs, Thermotogae, Calescamantes, Pyropristinus, hyperthermophiles, Yellowstone National Park

## Abstract

Thermal spring ecosystems are a valuable resource for the discovery of novel hyperthermophilic *Bacteria* and *Archaea*, and harbor deeply-branching lineages that provide insight regarding the nature of early microbial life. We characterized bacterial populations in two circumneutral (pH ~8) Yellowstone National Park thermal (*T* ~80°C) spring filamentous “streamer” communities using random metagenomic DNA sequence to investigate the metabolic potential of these novel populations. Four *de novo* assemblies representing three abundant, deeply-branching bacterial phylotypes were recovered. Analysis of conserved phylogenetic marker genes indicated that two of the phylotypes represent separate groups of an uncharacterized phylum (for which we propose the candidate phylum name “Pyropristinus”). The third new phylotype falls within the proposed Calescamantes phylum. Metabolic reconstructions of the “Pyropristinus” and Calescamantes populations showed that these organisms appear to be chemoorganoheterotrophs and have the genomic potential for aerobic respiration and oxidative phosphorylation via archaeal-like V-type, and bacterial F-type ATPases, respectively. A survey of similar phylotypes (>97% nt identity) within 16S rRNA gene datasets suggest that the newly described organisms are restricted to terrestrial thermal springs ranging from 70 to 90°C and pH values of ~7–9. The characterization of these lineages is important for understanding the diversity of deeply-branching bacterial phyla, and their functional role in high-temperature circumneutral “streamer” communities.

## Introduction

The discovery and characterization of early-branching lineages of *Bacteria* and *Archaea* has been crucial for studying the origin and evolution of life on Earth. There is considerable evidence for the hypothesis that life originated in environments similar to modern hydrothermal settings, although other scenarios are also proposed (e.g., cold origins; Price, 2009). Hyperthermophiles inhabit geothermal environments that are analogous to those of early Earth (Baross and Hoffman, 1985), and are generally the deepest branching representatives of the tree of Life (Di Giulio, 2003; Stetter, 2006). The well-characterized and largely hyperthermophilic bacterial phyla Aquificae and Thermotogae have been considered the most basal bacterial lineages on the basis of phylogenetic evidence (Barion et al., 2007; Zhaxybayeva et al., 2009). More recently, an uncultured bacterium from subsurface thermal fluids, *Candidatus* “Acetothermum autotrophicum,” has also been posited as one of the deep lineages in the *Bacteria* based on phylogenetic analysis of genome sequence (Takami et al., 2012). Consequently, discovery and characterization of new and uncultured lineages of thermophilic microorganisms are extremely useful toward the broader goal of understanding genomic and metabolic attributes of deep-branching phyla, which inhabit modern-day environments that may be analogs to those potentially important in the origin(s) of life.

The characterization of uncultured microorganisms from thermal environments has been integral for expanding the scope of known microbial diversity. Early phylogenetic surveys based on 16S rRNA gene analysis revealed a significant diversity of uncultivated microorganisms in various hydrothermal settings, including numerous candidate phyla (Barns et al., 1994; Reysenbach et al., 1994; Hugenholtz et al., 1998; Takai and Horikoshi, 1999). However, due to the difficulty of cultivating environmentally relevant microorganisms (particularly extremophiles), the physiological diversity of many of these phyla has remained largely unknown since their discovery. Environmental genomics (e.g., metagenomics and single-cell genomics) has provided valuable tools for assessing the metabolic capabilities and phylogenetic diversity of thermophiles and other extremophilic *Bacteria* and *Archaea* (Baker et al., 2010; Nunoura et al., 2011; Takami et al., 2012; Dodsworth et al., 2013; Inskeep et al., 2013; Kantor et al., 2013; Kozubal et al., 2013; Rinke et al., 2013; Hedlund et al., 2014; Wrighton et al., 2014; Castelle et al., 2015). However, numerous microbial phyla remain uncharacterized, and continued studies in high-temperature habitats hold promise for dissecting the functional role of early-branching lineages in less-complex microbial communities.

Filamentous “streamer” communities containing members of the Aquificales are common in geothermal spring outflow channels and hydrothermal vents in marine systems globally (Ferrera et al., 2007). We recently described and characterized metagenomes from six filamentous “streamer” communities from geochemically distinct habitat types from Yellowstone National Park (YNP; Inskeep et al., 2013; Takacs-Vesbach et al., 2013). Three primary genera of Aquificales dominate different streamer communities based on geochemical conditions (e.g., pH, sulfide), and each habitat type supports different co-occurring heterotrophic community members. Two non-sulfidic, slightly alkaline (~7.8–8) streamer communities (Octopus and Bechler springs) contained abundant *Thermocrinis* spp. (Aquificales) and representatives of several uncultured bacterial lineages. A novel member of the Aigarchaeota (*Ca*. Calditenuis aerorheumensis) was recently characterized from the Octopus Spring (OS) streamer communities (Beam et al., 2016); however, several novel and abundant bacteria in these communities have remained uncharacterized. Consequently, the objectives of this study were to (1) obtain and curate *de novo* sequence assemblies from these two streamer communities corresponding to three uncharacterized bacterial phylotypes, (2) assess the phylogenetic position and functional potential of the three phylotypes, and (3) determine the distribution of these populations in YNP and other thermal environments. Here we describe three new phylotypes curated from random shotgun Sanger sequencing of two slightly alkaline (pH ~8) filamentous “streamer” communities (temperature ~80°C) from Octopus and Bechler springs (Yellowstone National Park). These aerobic chemoorganoheterotrophs are representatives of two distinct and deeply-branching, phylum-level lineages in the domain *Bacteria*. “Pyropristinus” is proposed here as a newly described lineage containing two distinct phylotypes, while the other novel phylotype belongs to the proposed Calescamantes phylum (Rinke et al., 2013; Hedlund et al., 2014). The discovery and characterization of these early-branching bacteria are critical for dissecting microbial community structure and function in modern-day high-temperature habitats, and provides significant opportunities for understanding the evolution of deeply-branching hyperthermal bacterial lineages.

## Materials and methods

### Recovery of uncharacterized bacterial populations from hot-spring metagenomes

Details of site sampling, metagenome sequencing, assembly, and geochemical analyses have been described previously (Inskeep et al., 2013; Takacs-Vesbach et al., 2013). Briefly, filamentous microbial community samples were collected near the top of the spring runoff channels from a spring in the Bechler Three Rivers Junction region of YNP (*T* = 80–82°C, pH = 7.8; 44.2859 N, −110.8784 E) and Octopus Spring in the Lower Geyser Basin of YNP (*T* = 80–82°C, pH = 7.9; 44.53408 N, −110.7979 E). A phenol/chloroform extraction method was used to extract community DNA (Inskeep et al., 2010), which was then used to construct a small-insert clone library. Sanger sequencing was used for random shotgun sequencing of the inserts (~40 Mb total DNA sequence for each site). Metagenomes were assembled using the Celera assembler; automated tools in the Integrated Microbial Genomes server (IMG; Markowitz et al., 2012) were used to predict and annotate genes. Nucleotide word frequency-principal components analysis (NWF-PCA) was used to identify predominant populations in the metagenomic contigs (>3 kbp) as described previously (Takacs-Vesbach et al., 2013). The contigs were further analyzed and screened using G+C content (%) and phylogenetic analysis (most useful for phylotypes exhibiting closest neighbors above 80% nt ID) to obtain four *de novo* sequence assemblies corresponding to abundant and uncharacterized members of these communities (T1.1, T1.2, T2.1, T3.1; contig coverage > 1). Genome completeness was estimated using three metrics: tRNA synthetase complement was estimated by the presence of at minimum, one partial annotated gene for each of 21 prokaryotic genes coding for tRNA synthetases, the presence of 178 “conserved” bacterial housekeeping genes (Garcia Martin et al., 2006) and 40 “conserved” prokaryotic universal markers (Wu et al., 2013).

Amino acid identity (AAI) was used to assess taxonomic relationships among assemblies and other closely related genomes. AAI was calculated as the mean percentage of differing amino acid residues between homologous protein coding genes in pairwise comparisons of assemblies using blastp after filtering of low quality alignments (Konstantinidis and Tiedje, 2005b). Protein coding gene alignments were used that (1) shared at least 30% a.a. similarity, (2) were alignable up to 70% of the length of the subject sequence, and (3) had an alignment length of at least 100 residues. The T3.1 (Calescamantes-like) phylotype from OS was also compared to the recently described *Ca*. Calescibacterium nevadense (EM19-like) genome (IMG taxon ID: 2527291514). Average nucleotide identity (ANI) was calculated between scaffolds of the two closely related assemblies, T1.1 and T1.2 using default parameters (minimum length = 700 bp, window size = 100 bp, min. identity = 70%, min alignment number = 50, window size = 100 bp and step size = 200 bp) with the online ANI calculator (http://enve-omics.ce.gatech.edu/ani/index; Goris et al., 2007).

### Phylogenetic analyses

Phylogenetic analyses were conducted by surveying all three lineages (T1, T2, and T3) for homologous single-copy housekeeping genes (at least partial copies shared amongst all three lineages) that were previously identified as bacterial-specific or universal (Wu et al., 2013). Genomic references were chosen based on blastp searches of ribosomal proteins against publically available genomes and curated such that every bacterial reference (Supplementary Table 1) contained ≥ 16 of the 18 total genes (5 universal: *pheT, rplE, rplO, rpsK, rpsM*, and 13 bacterial-specific marker genes: *clpX, leuS, ligA, murD, pnp, pth, pyrG, rplL, rpoA, rpoB, rpoC, secY, serS;* with the exception of *Ca*. C. nevadense: 3 genes missing). Each gene was aligned individually with Clustal Omega (Sievers et al., 2011), and alignment positions were confidence weighted using Zorro (Wu et al., 2012) to reduce the influence of ambiguously aligned positions. An evolutionary substitution model was chosen for each individual gene alignment using ProtTest v. 3.4 (Darriba et al., 2011). The concatenated gene alignment (8928 informative amino acid positions) was used in a maximum likelihood (ML) analysis in RAxML v. 8.2.3 (Stamatakis, 2006) using alignment weights, and partitioning the concatenation so that each gene was modeled separately by the appropriate substitution model (primarily the LG substitution model; with a gamma distribution of rates and proportion of invariant sites). Archaeal outgroups (*n* = 27) were used to root bacterial phylogenies using the five universal single copy housekeeping genes of the dataset that were common to both *Bacteria* and *Archaea*. Phylogenies were bootstrapped with 1000 ML replicates using the RAxML rapid bootstrapping algorithm.

Phylogenetic analysis was also conducted using near full-length 16S rRNA genes (>1300 bp). The T3.1 (EM19-like) and T1.1 assemblies were omitted from this analysis because they did not contain full-length 16S rRNA genes, although conspecific (>97% nt identity) relatives of these lineages serve as proxies for their phylogenetic placement. A 953 bp 16S rRNA gene present in the Octopus Spring metagenome (but not included in the T3 *de novo* assembly due to length) was 99% identical (nucleotide) to a nearly full length EM19-like 16S rRNA gene sequence obtained from Octopus Spring (OS_clone_YNP11_11_1). Further, the ~850 bp T1.1 16S rRNA gene from the T1.1 Octopus Spring assembly was 98% identical to the nearly full-length T1.2 16S rRNA gene from the Bechler assembly, and thus the more complete T1.2 16S rRNA gene sequence from Bechler was used to represent both the OS T1.1 and the Bechler T1.2 population. Genes were aligned using PyNAST (Caporaso et al., 2010) with the Greengenes reference dataset (DeSantis et al., 2006). The DNA substitution model for the alignment was selected using Modeltest v. 3.7 (Posada and Crandall, 1998) and the Akaike Information Criterion (AIC) model metric. ML analysis was conducted in MEGA v.6 (Tamura et al., 2013) using the General Time Reversible model with a proportion of invariant sites and a gamma distribution of rates.

Conserved signature indel (CSI) analyses were also used to assess if the newly described lineages belonged to closely related phyla, such as the Thermotogae and Aquificae. CSIs specific to the Thermotogae (18 total; Gupta and Bhandari, 2011) or Aquificae (4 total; Gupta and Lali, 2013), relative to the rest of *Bacteria*, were used by referencing the *de novo* sequence assemblies against available Thermotogae and Aquificae genomes available in IMG. A total of 22 genes (encompassing 22 CSIs) were aligned with Clustal, as described in the original study (Gupta and Bhandari, 2011), and inspected for the characteristic CSIs.

### Metabolic reconstruction

Functional similarity of the “Pyropristinus” and Calescamantes-OS types relative to the Thermotogae, Aquificae, *Ca*. Calescibacterium nevadense, and Thermodesulfobacteria were statistically assessed with a non-metric multidimensional scaling (NMDS) ordination of the presence-absence of COGs in each genome (using Euclidean distances) in R. Annotated genes were used to assess the presence of metabolic pathways in all three lineages. The conspecific-level relatedness between T1.1 and T1.2 assemblies allowed the use of the less complete T1.1 assembly to augment the genes not found in T1.2. Where possible, genome sequence of *Ca*. Calescibacterium nevadense (Rinke et al., 2013) was used as a reference for the presence of pathways in T3, which was related to *Ca*. C. nevadense. Genomic data for the four assemblies produced here is available under the NCBI Bioproject ID PRJNA280379.

### Ecological distribution

Full-length 16S rRNA genes of the three lineages were used in BLASTn searches against available datasets to determine the habitat distribution of these newly described populations. Because a full-length 16S rRNA gene was not present in the T3.1 assembly, a representative sequence from the 16S rRNA gene library of the same Octopus Spring metagenome sample was used (Takacs-Vesbach et al., 2013). This clone group (EM19) was also described in pink-streamer communities of the same spring (Reysenbach et al., 1994; Blank et al., 2002). *Ca*. C. nevadense is also closely related to the EM19 clone from Octopus Spring (Rinke et al., 2013), and was the closest genome sequence available for comparison to the Calescamantes-like assembly from Octopus Spring (Calescamantes-OS; T3.1 used here). Searches were conducted against 16S rRNA gene datasets including Genbank, IMG metagenomes, Greengenes (DeSantis et al., 2006), the Ribosomal Database Project (Cole et al., 2014), as well as YNP-specific surveys (including 454 pyrosequencing datasets) of 49 YNP springs spanning a wide range of temperature and pH values (Takacs-Vesbach et al., unpublished data) and clone-libraries of 82 YNP springs (Mitchell, 2009). 16S rRNA gene matches with >97% nucleotide identity to each of the three lineages were considered a positive occurrence. Metadata for each reference sample (temperature, pH, and geographic location) were collected from the publishing reports, where available, and augmented with data from the YNP Research Coordination Network database (http://www.rcn.montana.edu; Supplementary Table 2). Mean values for sample temperature are used where ranges were reported. Statistical differences of temperature and pH distributions among groups were tested using a Kruskal-Wallis rank sum analysis of variance test in R (R Core Team, 2014).

## Results and discussion

### Recovery of uncharacterized bacterial populations from hot-spring metagenomes

**Figure 1:**
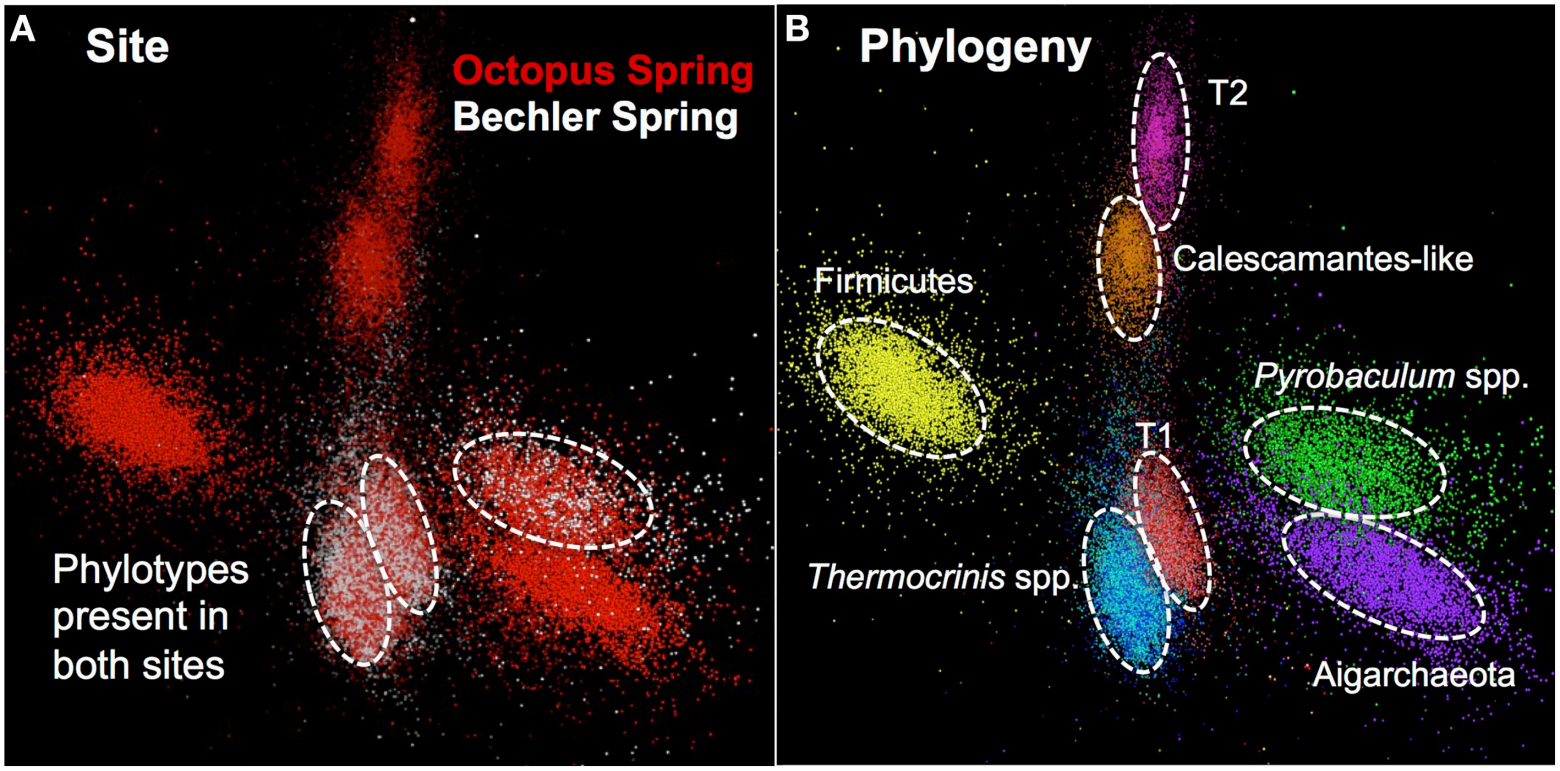
**Nucleotide word frequency PCA plots of metagenome assemblies from two Aquificales “streamer” communities in YNP**. **(A)** Data colored by site: Octopus Spring = red; Bechler spring = white. **(B)** Identical PCA orientation with phylogenetic analysis and assignment (dashed-white circles): “Pyropristinus” Type 1-r01 = red; “Pyropristinus” Type 1-r02 = light-red; “Pyropristinus” Type 2-r01 = pink; Calescamantes-like = orange; Firm_T1-r01 = yellow; *Thermocrinis*-r01 = dark-blue; *Thermocrin*is-r02 = light-blue; *Pyrobaculum* spp. = green; Aigarchaeota_T1-r01 = purple.

**Figure 2:**
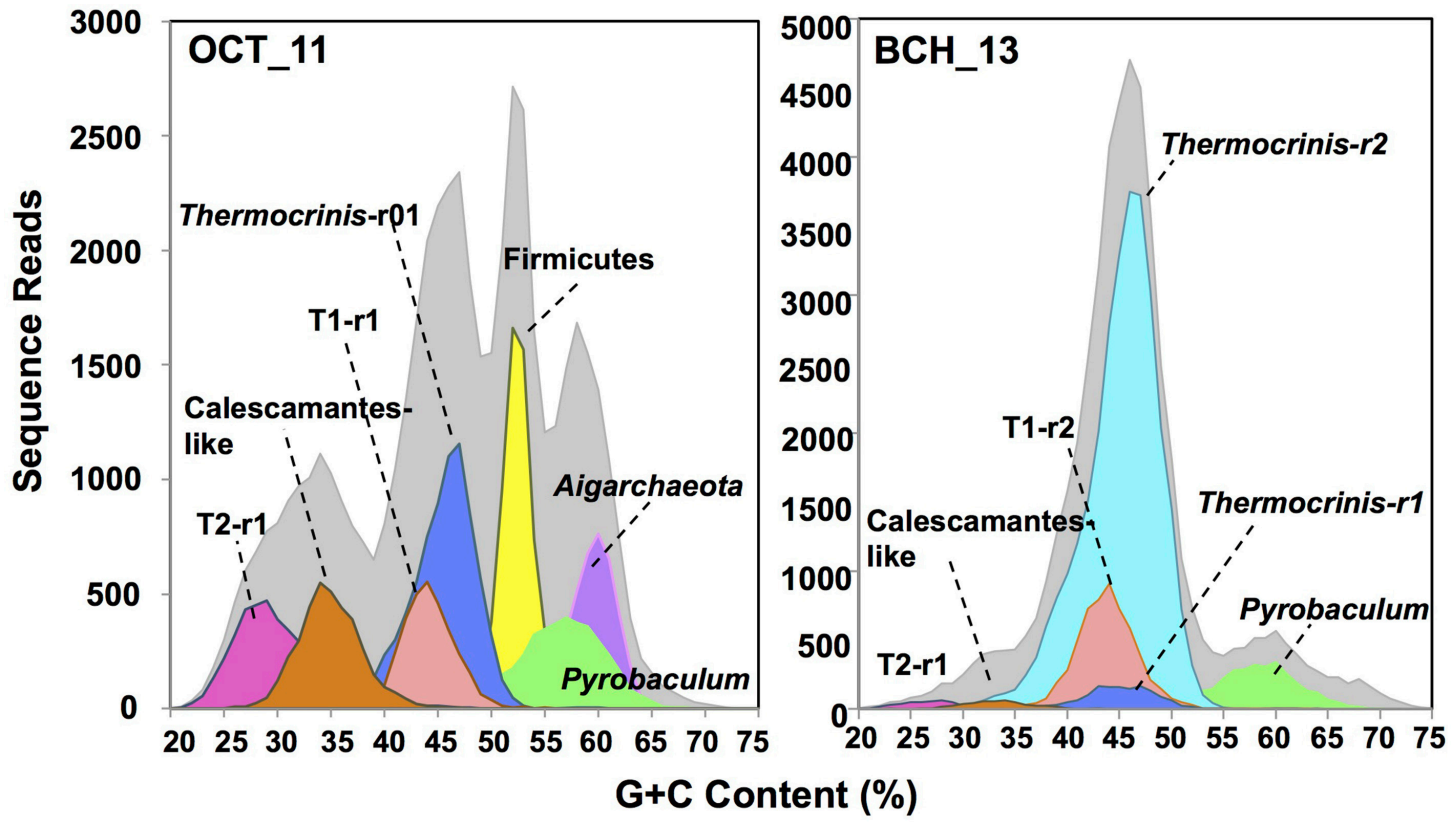
**Frequency plots of the G+C content (%) of random shotgun sequence reads (Sanger) from filamentous “streamer” communities at Octopus Spring (OCT_11) and Bechler springs (BCH_13)**. Taxonomic (phylogenetic) assignment of each sequence read was performed using BLASTn (>90% nt ID) against curated *de novo* assemblies generated from these sites (i.e., Figure 1): [light-gray = total reads, red = “Pyropristinus” T1-r1 (G+C = 44%), light-red = “Pyropristinus” T1-r02 (G+C = 44%), pink = “Pyropristinus” T2-r01 (G+C = 29%), orange = Calescamantes-like (G+C = 35%), blue = *Thermocrinis*-like r01 (G+C = 45.5%), light-blue = *Thermocrinis*-like r02 (G+C = 45%), yellow = *Firmicutes* (G+C = 53%), green = *Pyrobaculum*-like (G+C = 57–58%), purple = Aigarchaeota (G+C = 60%)].

The assembled metagenome sequence from Octopus and Bechler Springs was analyzed using nucleotide word frequency-principal components analysis (NWF-PCA) to obtain contigs and scaffolds (>3 kbp length only) sharing similar sequence character (Figure 1). These scaffolds and contigs were further separated using G+C content (%), coverage and phylogenetic analysis to obtain *de novo* sequence assemblies corresponding to each of the predominant phylotypes in these communities (Supplemental Figure 1). The Octopus Spring community contained at least eight predominant phylotypes (Desulfurococcales, not shown), while Bechler spring contained three abundant phylotypes (Figure 1A). Each of the two streamer communities contained highly-related populations of *Thermocrinis* spp. (Aquificales), *Pyrobaculum* spp. (Thermoproteales), and a novel population referred to here as Type 1 (T1) of candidate phylum “Pyropristinus.” The streamer community from Octopus Spring also contained abundant populations of a Type 2 (T2) “Pyropristinus” population, a relative of the proposed bacterial phylum Calescamantes (EM19 candidate division; Rinke et al., 2013; Hedlund et al., 2014), an uncharacterized member of the Firmicutes, and a member of the candidate archaeal phylum Aigarchaeota (Beam et al., 2016). The average coverage, G+C content (%), and cumulative sequence plots of contigs corresponding to the three “Pyropristinus” (T1.1, T1.2, T2), and the Calescamantes populations are provided in supplemental information (Supplemental Figure 1). To assess the contribution of these assemblies to population abundances in the metagenomic data, the *de novo* assemblies compiled from these sites were used to bin the original random metagenome sequence reads (Figure 2; Table 1). A G+C (%) frequency plot of random metagenome sequence reads (average read length = 820 bp) that were phylogenetically assigned (≥90% nucleotide identity) to the *de novo* assemblies compiled from these sites showed that “Pyropristinus” T1 and T2, as well as the Calescamantes-like phylotypes were significant members (~7–8% of all reads) of the more even microbial community in Octopus Spring, and that the “Pyropristinus” T1 was also abundant (~12%) in Bechler spring (Figure 2, Table 1).

**Table 1:** **Abundance estimates (%) of major population types^a^ obtained from Octopus and Bechler springs based on analysis of metagenome sequence reads against reference ***de novo*** assemblies (at 90% nucleotide identity)**.

**Phylotype**	**G+C (%)**	**Spring**	
		**Octopus**	**Bechler**
Aquificales, Thermocrinis spp.	45	18	62
Novel firmicutes	53	13	<1
“Pyropristinus” Type 1	44	8	12
“Pyropristinus” Type 2	29	8	<1
Aigarchaeota, Ca. Calditenuis aerorheumensis^b^	60	8	<1
Pyrobaculum spp.	57	7	7
Calescamantes-OS^c^	35	7	<1
Unknown bacteria		3	<1
Desulfurococcales	58	<1	<1
Orphans (Unassigned)		25	13

a *Populations shaded in gray are the focus of the current study*.

b *Beam et al., 2016*.

c *Hedlund et al., 2014*.

Average estimates of genome completeness based on housekeeping genes present in the *de novo* sequence assemblies were 65, 72, and 63% for the T1 “Pyropristinus” (T1.2), T2 Pyroprisitnus (T2.1), and the Calescamantes-like populations, respectively (Table 2). Estimates by tRNA synthetase complement were higher (86% for both T1.2 and T2) than those based on the presence of “conserved” housekeeping genes involved in many cellular processes (50–60% and 59–73% for T1.2 and T2, respectively). The lower estimates based on the presence of housekeeping genes involved in a variety of cellular processes may be conservative due to the lack of appropriate references for identifying these genes in phylogenetically novel, deep-branching organisms (discussed further below). The cumulative sequence and contig coverage plots, coupled with genome coverage's of ~2–3.5x and robustness of Sanger sequencing methodologies indicate that these genomes were adequately sampled (Supplemental Figure 1).

**Table 2:** **Genome assembly statistics for “Pyropristinus” Type 1 and Type 2, and Calescamantes populations from Octopus and/or Bechler springs**.

**Spring Name**	**“Pyropristinus” Type 1**	**“Pyropristinus” Type 1**	**“Pyropristinus” Type 2**	**Calescamantes-OS**
	**Octopus**	**Bechler**	**Octopus**	**Octopus**
Phyla Abbrev.	T1.1	T1.2	T2.1	T3.1
Size (in Mbp)	1.02	1.24	1.1	1.29
Completeness^a^	47.0	64.9	72.2	62.9
G+C (%)	44.3	44.3	28.9	35.2
Contigs	116	72	117	164
Coding genes	1249	1464	1376	1569
Longest contig (kbp)	34.4	59.2	32.5	26.1

a *Estimated based on the average of three completeness estimation methods: (1) tRNA aaRS complement; (2) 40 conserved universal prokaryotic housekeeping genes (Wu et al., 2013); (3) 178 conserved universal bacterial housekeeping genes (Garcia Martin et al., 2006)*.

### Phylogenetic analyses

**Figure 3:**
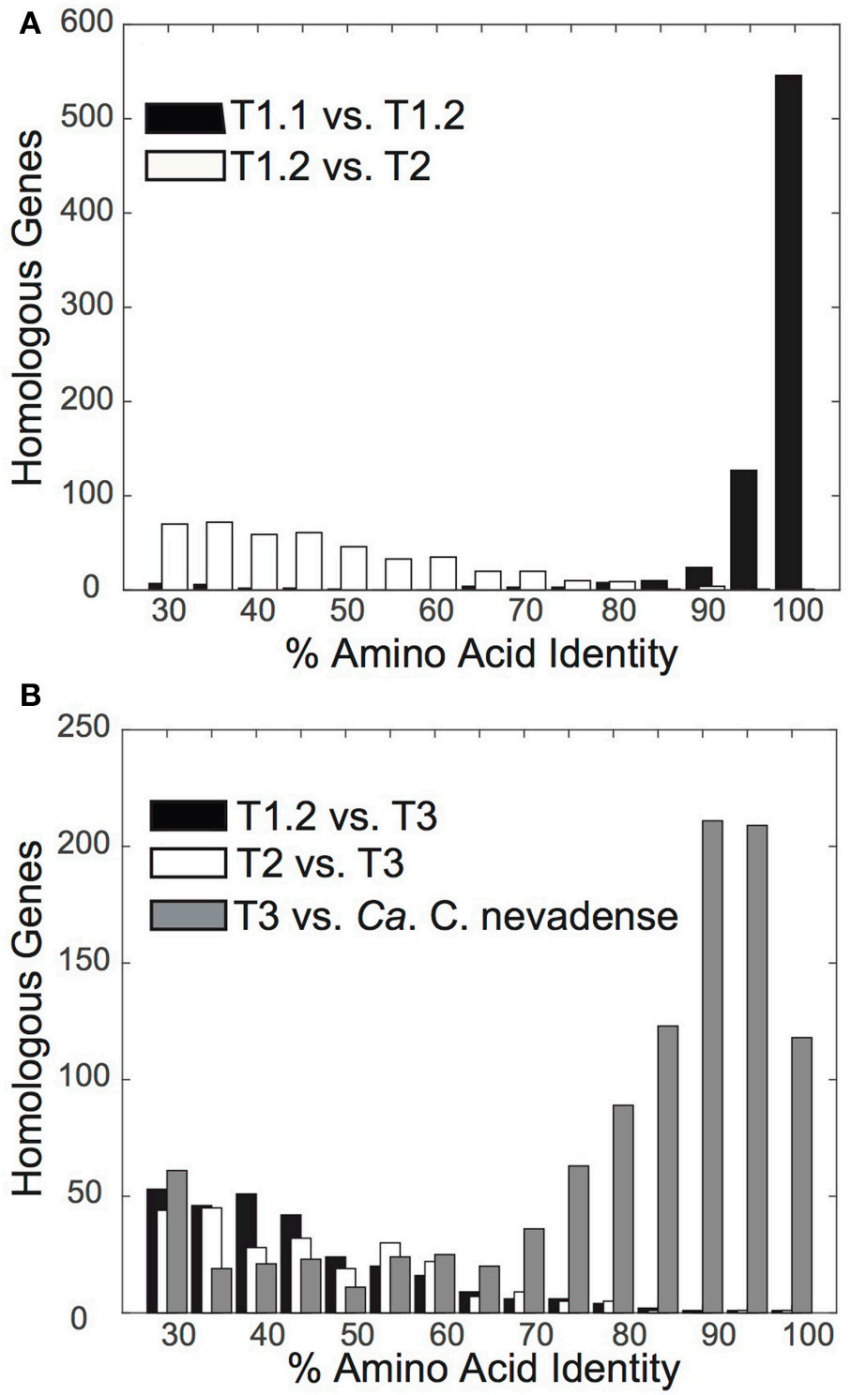
**Distribution plots of amino acid identity % (AAI) of protein-coding genes between pairwise comparisons of novel bacterial assemblies**. **(A)** Amino acid identity of “Pyropristinus” Type 1 from Octopus Spring (T1.1) vs. “Pyropristinus” Type 1 from Bechler Spring (T1.2) (black; mean = 94.2 ± 10.1%), and “Pyropristinus” Type 2 (T2.1 vs. T1.2) (white; mean = 46.6 ± 12.3%). **(B)** Amino acid identity of the Octopus Spring (OS) Calescamantes population vs. *Ca*. Calescibacterium nevadense (gray; mean = 78.0 ± 18.1%), “Pyropristinus” Type 1 (T1.2) (black; mean = 42.7 ± 10.0%), and “Pyropristinus” Type 2 (T2.1) (white; mean = 41.8 ± 9.0%).

The “Pyropristinus” T1 assemblies from Octopus and Bechler Springs (T1.1 and T1.2, respectively) were highly-related to one another independent of the comparison method: they exhibited ANIs of 96 ± 1.3% (*n* = 2410 comparisons), AAIs of 94 ± 10.1%, (homologous alignment *n* = 743; Figure 3A), and 16S rRNA gene identities of 98%. The high nucleotide and amino acid identities of the two “Pyropristinus” T1 assemblies indicate that these populations belong to the same genus and likely to the same species, based on empirical species delineations (Konstantinidis and Tiedje, 2005a,b). In contrast, the “Pyropristinus” T1.2 and T2 assemblies were substantially different from one another (AAI = 46.6 ± 12.3%, *n* = 442; Figure 3A), and both are considerably different than the Calescamantes-like population from Octopus Spring (average AAI ~42%; Figure 3B). While taxonomic rank delineations using AAI do not follow discrete cutoffs, an AAI of only 47% between T1 and T2 is consistent with phylum- or class-level differentiation (Konstantinidis and Tiedje, 2005b). Further, 16S rRNA genes from T1.2 and T2 only shared 84% similarity, which is consistent with empirical phylum- or class-level delineations (Yarza et al., 2014). Until more closely related genomic or isolate representatives are obtained to resolve the taxonomic differences between the T1 and T2-inclusive groups, we tentatively assign both phylotypes to the “Pyropristinus” division that we propose here. The Calescamantes population from OS (Calescamantes-OS) was more closely related to the recently described candidate species *Ca*. Calescibacterium nevadense from Great Boiling Spring, Nevada (78.0 ± 18.1% mean AAI, *n* = 1053; Figure 3B). The Calescamantes-OS and *Ca*. C. nevadense are different enough to suggest that these two populations represent different genera or even families within the proposed Calescamantes division (Rinke et al., 2013).

**Figure 4:**
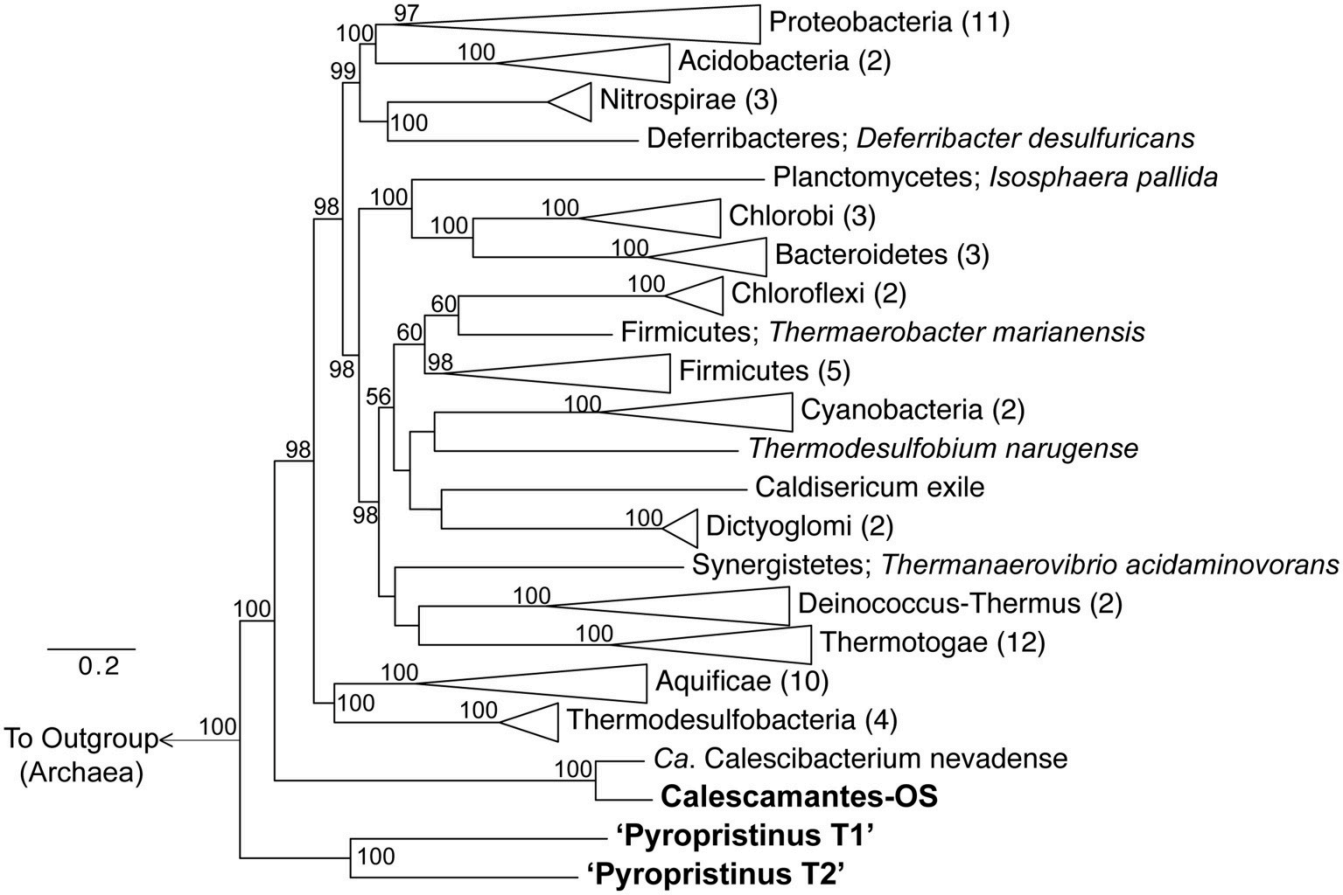
**Phylogenomic analysis of “Pyropristinus” and Calescamantes lineages**. Maximum-likelihood tree based on genomic analysis of 13 bacterial-specific and 5 universal housekeeping genes (total of 18 genes coding for 8928 amino acid positions). Twenty-seven archaeal references were used as an outgroup. Phyla with more than one reference were collapsed and the number of genomes per group are given in parentheses. Bootstrap values (1000 replicates) are given at the nodes where ≥50%. Scale shows expected substitutions per site.

Phylogenetic analyses using concatenations of 18 universal or bacterial-specific single-copy genes common to all three phylotypes (comprising 8928 informative amino acid positions) suggest that the “Pyropristinus” T1 and T2 lineages belong to a deeply-branching phylum, distinct from all currently characterized *Bacteria* (Figure 4). Analysis of only ribosomal proteins common to all three phylotypes (*rplE, rplL, rplO, rpsK, rpsM;* 788 informative amino acid alignment positions) confirmed that the “Pyropristinus” lineage is a well-supported monophyletic group, and is phylogenetically basal relative to the rest of *Bacteria* (Supplemental Figure 2). The phylogenomic analysis also confirmed that the “streamer” community from Octopus Spring contained a population related to the recently proposed Calescamantes phylum (formerly EM19; Rinke et al., 2013; Hedlund et al., 2014). Phylogenetic analysis without archaeal outgroups confirmed that the “Pyropristinus” and Calescamantes are highly supported groups separate from the Aquificae/Thermodesulfobacteria clade, and which together are distinct from all other *Bacteria* (Supplemental Figure 3).

**Figure 5:**
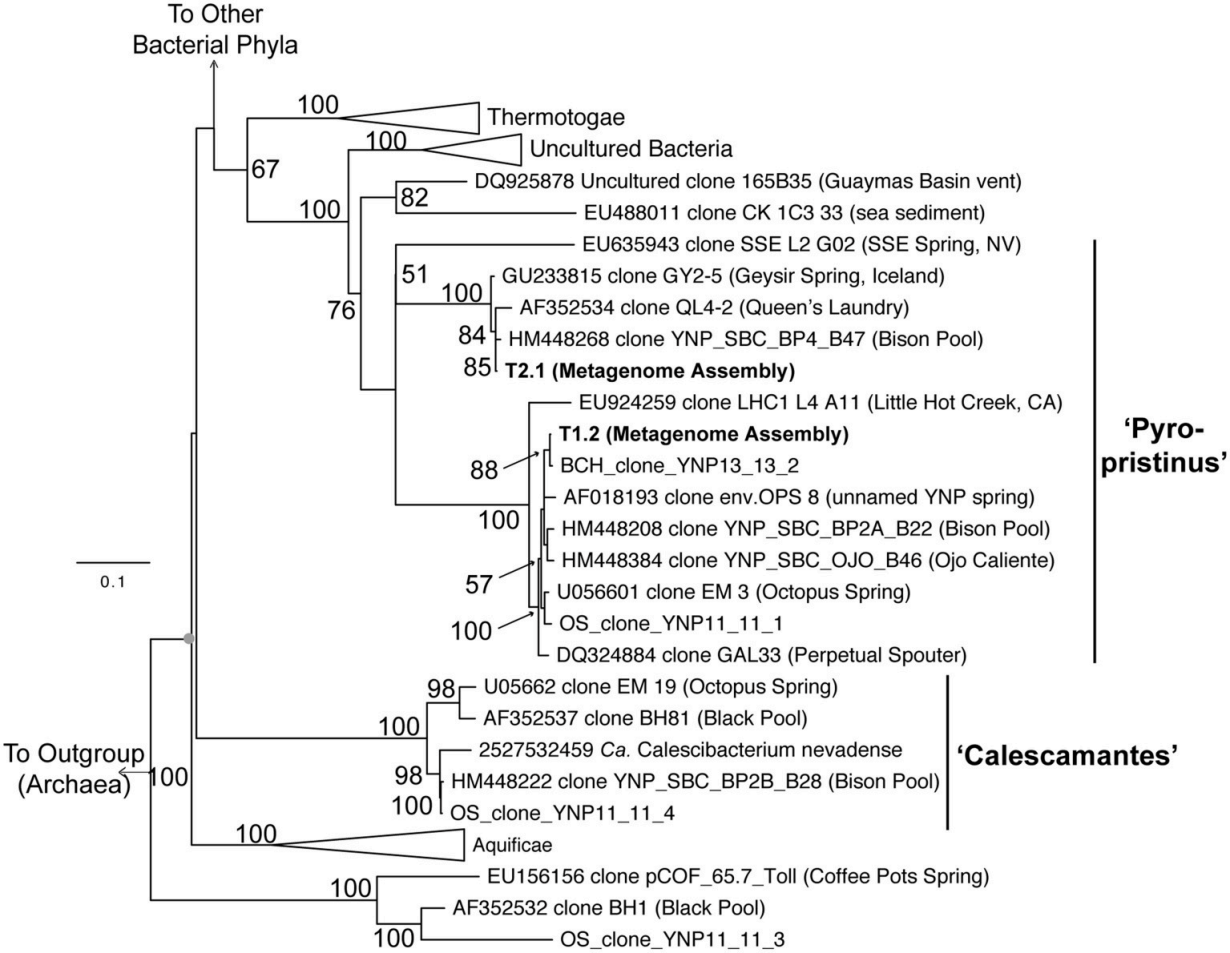
**Phylogenetic analysis using near full-length 16S rRNA genes**. 16S rRNA genes from the “Pyropristinus” Types T1 and T2 assemblies are indicated in bold (T1.1 and T2.1). The Calescamantes-OS assembly did not contain a full-length 16S rRNA gene and were thus omitted from this analysis). OSClone_YNP11_11_4, produced from a 16S rRNA gene library of the same Octopus Spring sample (also in bold) is nearly identical to the Calescamantes population from OS. Groups with multiple entries are collapsed as triangles. Bootstrap values (1000 replicates) are given at the nodes where ≥50%.

Phylogenetic analyses of these organisms using long-fragment 16S rRNA gene sequences also showed that both “Pyropristinus” T1 and T2 populations, along with other uncultured clones, form a deep-branching group near the Thermotogae (Figure 5). The “Pyropristinus” T1 population is closely-related (98% 16S rRNA gene identity) to the uncharacterized EM3 bacterium originally discovered in Octopus Spring (Reysenbach et al., 1994). Partial genome sequence for this “Pyropristinus” lineage was recovered from a single-cell from Great Boiling Spring, NV (only 14% estimated completeness by tRNA synthetase complement, IMG taxon ID: 2264867090; Rinke et al., 2013), but was not sufficiently complete for phylogenomic comparisons (e.g., Figure 4). The T1 and T2 lineages belonged to separate 16S rRNA gene clades, which is consistent with results from the phylogenomic comparisons. “Pyropristinus” T1 and T2 formed a cohesive group with other uncultured organisms from the same and/or similar types of hydrothermal systems (mean 16S rRNA gene distance within the group = 16%).

The “Pyropristinus” and Calescamantes populations lacked conserved signature indels (CSIs) typical of Thermotogae and Aquificae genomes, which is consistent with all other phylogenetic analysis showing that they are clearly separate from either of these characterized phyla (Supplementary Table 3). T1 shared two CSIs with the Aquificae, but neither were present in the “Pyropristinus” T2 or the Calescamantes-OS assemblies. However, the highly supported monophyletic grouping of the T1, T2, and Calescamantes-OS, which together were separate from the Aquificae in phylogenetic analyses, suggests that neither the “Pyropristinus” or Calescamantes groups are members of the Aquificae. Moreover, a comparison of homologous gene AAI of the “Pyropristinus” T1, T2, and Calescamantes-OS (T3) to several members of the Thermotogae (average AAI: T1/T2: 40.2%, T3: 39.8%, *n* = 5), Aquificae (T1/T2: 42.4%, T3: 42.6%, *n* = 4), and Thermodesulfobacteria (T1/T2: 42.0%, T3: 42.5%, *n* = 3) confirmed that the “Pyropristinus” lineages did not belong to the Thermotogae, the Aquificae, or the Thermodesulfobacteria, as was indicated in the phylogenomic analysis.

The relationship of the “Pyropristinus” and Calescamantes lineages to the recently described, deep-branching bacterium *Ca*. Acetothermum autotrophicum (Takami et al., 2012) was also attempted. However, due to a lack of universal housekeeping marker genes in the available sequence for *Ca*. “A. autotrophicum” (only three universal markers were shared among *Ca*. A. autotrophicum: IMG taxon ID: 2540341180, T1, and T2), consistent and well-supported placement of *Ca*. A. autotrophicum relative to the “Pyropristinus,” Calescamantes, Thermotogae, and Aquificae lineages could not be adequately assessed. A more robust set of universal marker genes from additional “Acetothermia” genome references will be necessary to confidently confirm the phylogenetic placement of “Acetothermia”-like populations.

### Metabolic reconstruction and potential community interactions

**Figure 6:**
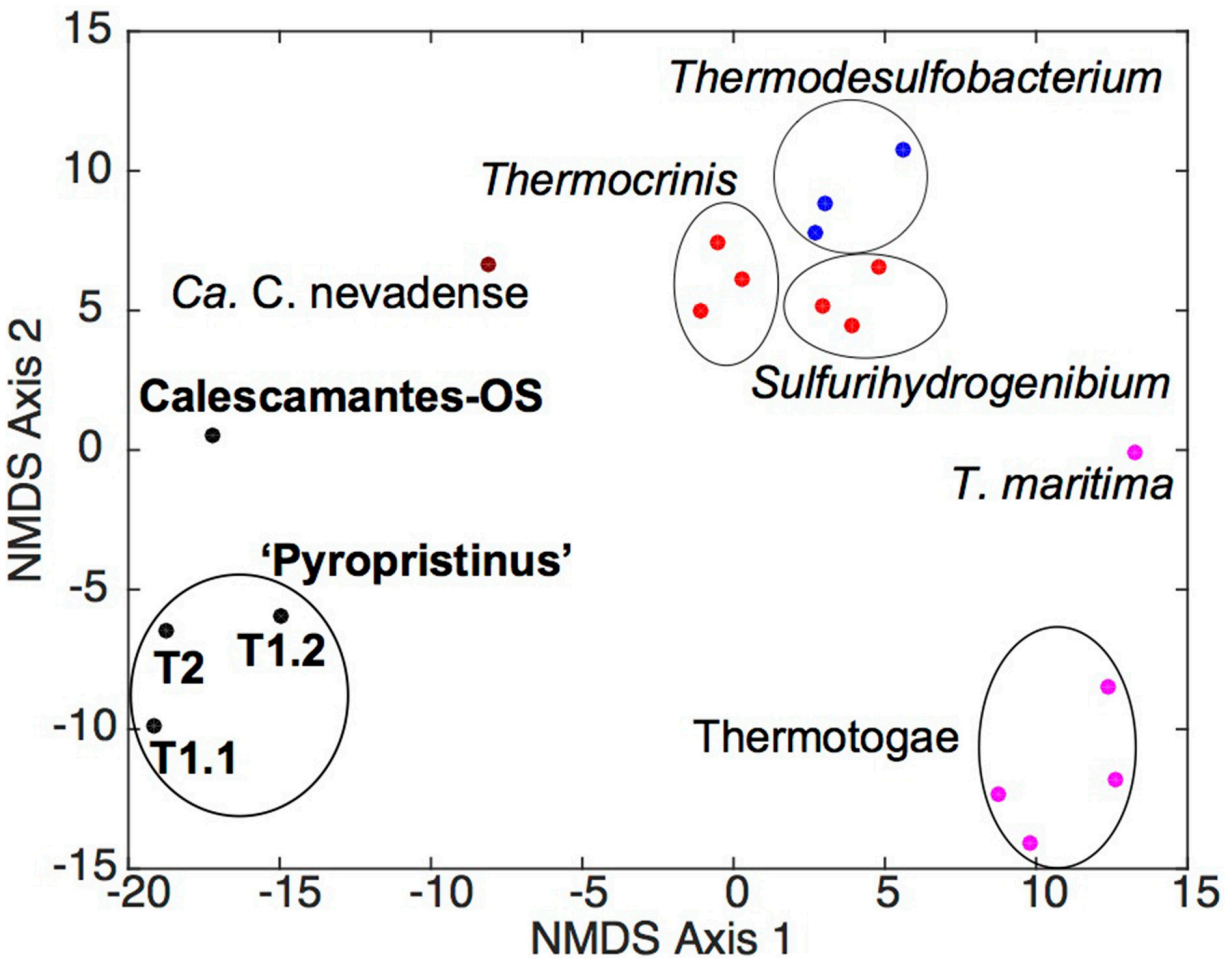
**Non-metric multidimensional scaling ordination plot of COG distribution among “Pyropristinus,” Calescamantes and closely related lineages**. NMDS plots (stress 0.08) were constructed from presence/absence Euclidean-distance matrices of COG groups present in the “Pyropristinus” T1 and T2 assemblies and Calescamantes-OS in addition to *Ca*. Calescibacterium nevadense (dark red), a subset of Aquificae (bright red), Thermodesulfobacteria (blue) and Thermotogae (purple) genomes, which comprised the closest related lineages to the “Pyropristinus” and Calescamantes lineages.

Metabolic reconstruction of the “Pyropristinus” T1 and T2 populations showed that these organisms shared nearly all major biochemical attributes, despite their substantial phylogenetic dissimilarity. Statistical analysis of the COG distributions from the “Pyropristinus” (T1 and T2) and Calescamantes populations indicated that the functional content of the T2 assembly was highly similar to the two T1 assemblies, and that the “Pyropristinus” assemblies were distinct from the Aquificae, Thermodesulfobacteria, and Thermotogae (Figure 6). The Calescamantes-OS population was also functionally distinct from the “Pyropristinus” assemblies, and was clearly separate from the *Ca*. C. nevadense assembly from Great Boiling Spring, Nevada.

#### Central carbon metabolism

**Figure 7:**
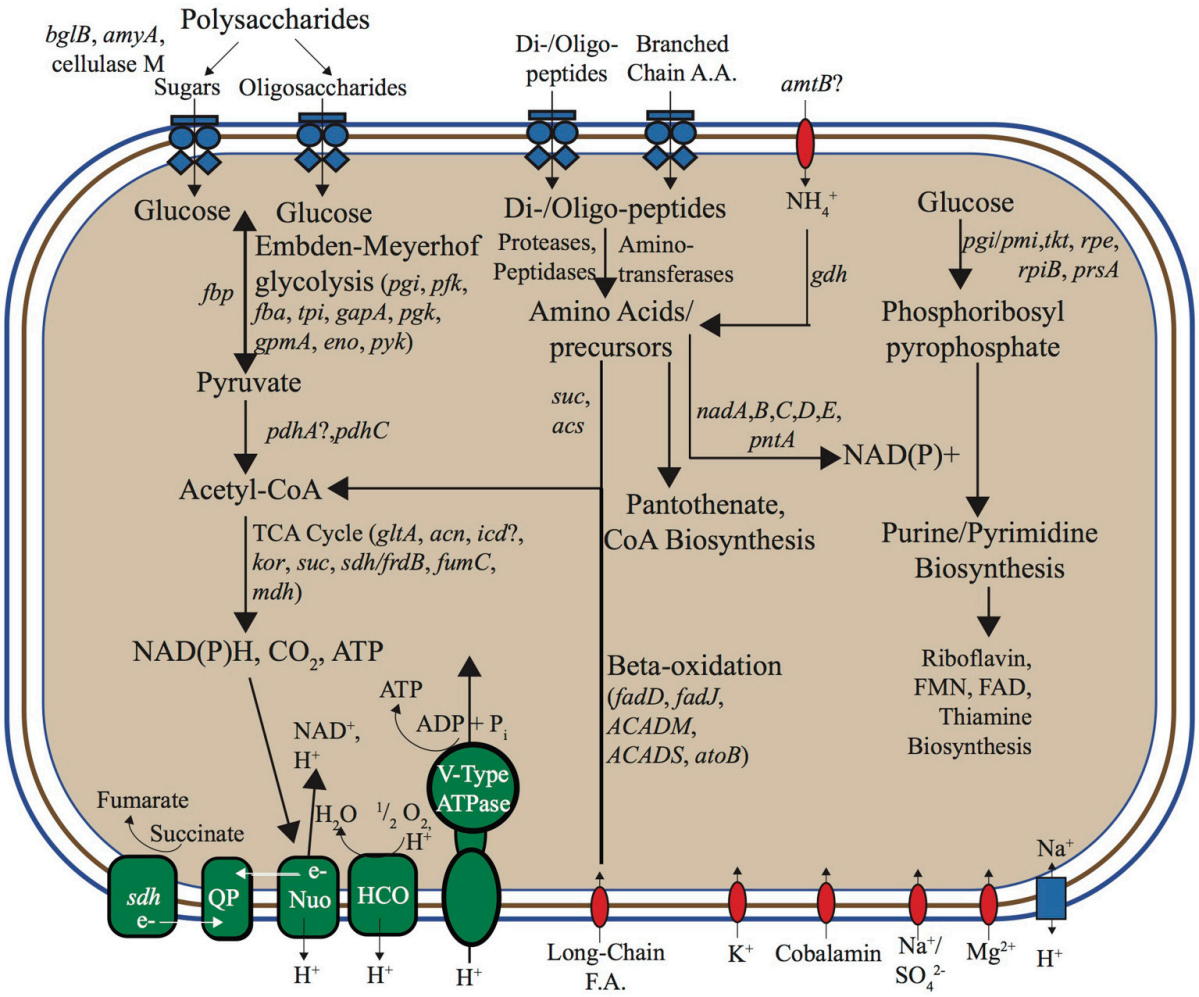
**Metabolic reconstruction based on annotation and manual curation of “Pyropristinus” Type 1 ***de novo*** assemblies**. ABC-type transporters are represented as blue multi-component transmembrane proteins, other transporters as red transmembrane proteins, and the single antiporter as a blue rectangle. Complexes and enzymes used in aerobic respiration/ATP synthesis are identified in green. Gene abbreviations: *bglB*, phospho-β-glucosidase; *amyA*, α-amylase; LPS, Lipopolysaccharides; *nadA*, quinolinate synthase, *nadB*, L-aspartate oxidase; *nadC*, quinolinate phosphoribosyltransferase; *nadD*, nicotinate-mononucleotide adenylyltransferase; *nadE*, NAD synthetase; *pntA*, pyridine nucleotide transhydrogenase (α subunit); *pgi*, phosphoglucose isomerase; *pfk*, 6-phosphofructokinase I; *fba*/*p*, fructose bisphosphate aldolase/phosphatase; *tpi*, triose phosphate isomerase; *gapA*, glyceraldehyde 3-phosphate dehydrogenase-A complex; *pgk*, phosphoglycerate kinase; *gpmA*, 2,3-bisphosphoglycerate-dependent phosphoglycerate mutase; *eno*, enolase; *pyk*, pyruvate kinase; *pdhA*, pyruvate dehydrogenase (lipoamide); *pdhC*, pyruvate dehydrogenase E2 component; *gltA*, citrate synthase; *acn*, aconitate hydratase; *icd*, isocitrate dehydrogenase; *kor*, 2-oxoglutarate:ferredoxin oxidoreductase; *suc*, succinyl-CoA synthetase, *sdh*, succinate dehydrogenase; *frdB*, fumarate reductase iron-sulfur protein; *fumC*, fumarase C; *mdh*, malate dehydrogenase; *amtB*, ammonium transporter; *gdh*, glutamate dehydrogenase; *acs*, acetyl-CoA synthetase; *fadD*, fatty acyl-CoA synthetase; *fadJ*, 3-hydroxyacyl-CoA dehydrogenase; *ACADM*, acyl-CoA dehydrogenase (C-4 to C-12); *ACADS*, acyl-CoA dehydrogenase (C-2 to C-3); *atoB*, acetyl-CoA acetyltransferase; QP, quinone pool; *nuo*, NADH:ubiquinone oxidoreductose complex; HCO, Heme-Cu oxidase; *pgi*/*pmi*, glucose/mannose-6-phosphate isomerase; *tkt*, transketolase; *rpe*, ribulose-5-phosphate 3-epimerase; *rpiB*, ribose-5-phosphate isomerase B; *prsA*, ribose-phosphate pyrophosphokinase; FMN, flavin mononucleotide; FAD, flavin adenine dinucleotide. Question marks indicate genes not identified in either Type 1 assembly (list of identified protein-coding genes in Supplementary Table 4).

No evidence for inorganic C fixation (Fuchs, 2011) was found in either the “Pyropristinus” (T1 and T2) or Calescamantes-OS populations, which suggests that these organisms are heterotrophic (Figure 7). The lack of CO_2_ fixation pathways in the Calescamantes-OS is consistent with analysis of the related *Ca*. C. nevadense (Hedlund et al., 2014). The metabolism of polysaccharides was indicated in the “Pyropristinus” (T1, T2) and Calescamantes lineages by the presence of β-glucosidases and α-amylases, as well as other important protein-coding genes in polysaccharide degradation (cellulase in T1; α-glucosidase and starch synthase in Calescamantes). An oligosaccharide transporter present in T1 also suggests that they may utilize exogenous saccharides produced by autotrophic streamer community members, such as *Thermocrinis* spp. (Aquificales), and/or Aigarchaeota that are also present in these communities (Takacs-Vesbach et al., 2013; Beam et al., 2016). All genes necessary for Embden-Meyerhoff glycolysis were present in “Pyropristinus” T1 (Figure 7), and most were also present in the Calescamantes population (and *Ca*. C. nevadense) indicating the potential to oxidize glucose. The presence of an archaeal-like fructose 1,6-bisphosphatase (*fbp*) also indicated that gluconeogenesis may occur via a bacterial variant of the bifunctional enzyme that is conserved in *Archaea* and early-branching bacterial lineages such as the Aquificae (Say and Fuchs, 2010). A nearly complete oxidative TCA cycle was also present in T1 (exclusive of *idh*) and both Calescamantes populations (Calescamantes-OS and *Ca*. C. nevadense). Both the “Pyropristinus” and Calescamantes groups contained protein-coding genes involved in the oxidation of fatty acids to acetyl-CoA (β-oxidation pathway; Figure 7). Moreover, long-chain fatty acid transporters present in “Pyropristinus” assemblies may indicate heterotrophic dependence on fatty acids from other streamer community members. The “Pyropristinus” T1 and T2 populations did not contain any evidence of anaerobic fermentation, such as alcohol dehydrogenases, acetate kinases, formate dehydrogenases, and/or [FeFe] or [NiFe] hydrogenases. However, alcohol dehydrogenases were present in the Calescamantes group (both the OS and *Ca*. C. nevadense assemblies), which suggests possible fermentation in these phylotypes. The “Pyropristinus” and Calescamantes populations contained several amino acid/peptide transporters, peptidases and proteases, which further suggests the ability to import oligopeptides and/or amino acids that may be present in the streamer microenvironment for heterotrophic metabolism.

The *Thermocrinis* spp. that are abundant in both Octopus and Bechler springs are capable of autotrophy via the reverse TCA carbon fixation pathway (Takacs-Vesbach et al., 2013) and are thought to be the primary producers in streamer filament communities (Takacs-Vesbach et al., 2013). Accordingly, the other co-occurring streamer community members, including a *Pyrobaculum* sp. and *Ca*. Calditenuis aerorheumensis, are predominantly, or strictly heterotrophic (Jay et al., 2015; Beam et al., 2016). The presence of heterotrophic “Pyropristinus” and Calescamantes-like organisms in these streamer communities provides further evidence for the hypothesis that the dominant *Thermocrinis* spp. support a diversity of co-occurring heterotrophic streamer community members. Moreover, C isotope studies have shown a mixture of both autotrophy and heterotrophy in streamer communities (including OS spring), and that these community members are capable of responding to transient organic carbon pulses (Schubotz et al., 2013; Jennings, 2015; Urschel et al., 2015). Thus, the “Pyropristinus” and Calescamantes-like organisms present in the streamer communities are likely utilizing both endogenous and exogenous organic carbon sources for heterotrophic metabolism.

#### Energy conservation

Nearly complete respiratory complexes including subunit I heme Cu oxidases were recovered in the “Pyropristinus” T1 and T2 populations as well as the Calescamantes representatives, which indicate that these organisms likely utilize oxygen for respiration and conduct oxidative phosphorylation (Figure 7). The “Pyropristinus” T1 lineage and Calescamantes (OS/*Ca*. C. nevadense) assemblies contained nearly complete NADH:quinone oxidoreductase (*nuo*) complexes necessary for NADH-mediated oxidative phosphorylation (Figure 7), but differed significantly in key energy conservation mechanisms. The “Pyropristinus” (T1/T2) assemblies contain archaeal V (vacuolar)-type ATPases, while the Calescamantes assembly contains a nearly complete F_0_F_1_ F-type ATPase. Only a small number of bacteria contain archaeal V-type ATPases; the F-type ATPase is ubiquitous and phylogenetically conserved among *Bacteria*, and is thought to be the ancestral bacterial ATPase (Mulkidjanian et al., 2007). The Thermotogae variously contain V-type or F-type ATPases (Nelson et al., 1999; Iida et al., 2002; Nesbo et al., 2002), whereas the Aquificae contain F-type ATPases (Koumandou and Kossida, 2014). The recently described deep-branching bacterium, *Ca*. Acetothermum autotrophicum, also contains an archaeal V-type ATPase (Takami et al., 2012). A BLAST search of an ATPase subunit I protein (614 aa) of the T1.2 assembly against available genomes in the IMG database showed limited homology (<33% aa id) to 11 genomes largely within the Synergistetes phylum, and to a lesser extent, the Firmicutes, Deltaproteobacteria and Actinobacteria. A BLAST search of an ATPase subunit I fragment present in the T2.1 assembly (233 bp; 40% homology to T1.2), showed similarly low homology to the above bacteria in addition to methanogenic archaea, Thermoplasmatales, and Archaeoglobi (<35% aa id). The disparity in archaeal-like and bacterial-like ATPase complexes between the “Pyropristinus,” Thermotogae, and *Ca*. A. autotrophicum lineages relative to the Calescamantes, Aquificae, and other bacteria suggests a major divergence in energy conserving mechanisms among these lineages, and warrants further investigation.

Key genes involved in ${\text{NH}}_{4}^{+}$ oxidation (*amo*), sulfur oxidation (*sqr, hdr, tqo*), sulfur/sulfate reduction (*psr, dsr*), H_2_ oxidation (*hyn*), methanotrophy (*pmo*), arsenate/arsenite metabolism (*arr, aox*), and ${\text{NO}}_{3}^{-}$ reduction (*nar, nap*) were not present in either of the “Pyropristinus” or the Calescamantes-OS populations. A *sqr*-like gene in the *Ca*. C. nevadense assembly suggests that HS^−^ may serve as an electron donor in that phylotype, however, a homologous *sqr* was not found in the Calescamantes-OS assembly. A nitrite reductase (*nirS*) present in *Ca*. C. nevadense with high homology (70%) to the cytochrome *cd*_1_ nitrite reductase from *Hydrogenobacter thermophilus* TK-6 (Aquificales; Suzuki et al., 2006) along with *nosZ* nitrous oxide reductase genes in the OS and *Ca*. C. nevadense assemblies suggests the potential for dissimilatory nitrite reduction in these organisms. However, as is common in the assembly of *de novo* genomes from environmental communities, the assemblies were not entirely complete (discussed above) and further analyses are needed to confirm the absence of genes necessary for lithotrophy or anaerobic respiration.

#### Secondary metabolites

Several differences among the “Pyropristinus” and Calescamantes populations were also noted in pathways used for the synthesis of secondary metabolites. For example, vitamin B_12_ is a necessary cofactor for methylmalonyl-CoA mutase (*mcm*), which is present in “Pyropristinus” and Calescamantes, and is important in the degradation of amino acids and fatty acids into succinyl-CoA (Martens et al., 2002). “Pyropristinus” populations lacked all genes necessary for the synthesis of cobalamin (Vitamin B_12_), whereas the Calescamantes-OS populations contained only 33% of the ~30 genes necessary for *de novo* synthesis (similar to *Ca*. C. nevadense, which contained ~53% of these genes). “Pyropristinus” (T1 and T2) populations contained genes coding for outer-membrane cobalamin receptor proteins and a permease involved in cobalamin transport, which suggests that they import this cofactor from the environment. The presence of biotin synthase (*bioB*) as well as *bioADF* suggest that the Calescamantes populations are capable of synthesizing biotin; conversely, the presence of only one biotin synthesis gene (*bioH*) from the “Pyropristinus” lineages suggests biotin auxotrophy. All of the assemblies contain acetyl-CoA carboxylases, which require biotin for the synthesis of malonyl-CoA from acetyl-CoA in fatty acid synthesis (Streit and Entcheva, 2003), and further supports a divergence in secondary metabolite acquisition between “Pyropristinus” and the Calescamantes.

The “Pyropristinus” assemblies lacked all genes necessary for flagellar synthesis, whereas the Calescamantes populations contained numerous flagellar biosynthesis genes including *flhA, fliM, fliN, fliE*, and *flgC*. The *Ca*. C. nevadense genome contained many of the flagellar biosynthesis genes not observed in the Calescamantes-OS population, and suggests that they are both capable of flagellar-mediated motility. Chemotaxis genes *cheY* and *cheD* were present in the Calescamantes-OS population, whereas “Pyropristinus” T1 contained *cheB, cheY*, and *cheC*. Both the Calescamantes and “Pyropristinus” populations are likely gram negative based on the presence of the essential outer-membrane protein assembly gene *yfiO* in Calescamantes, the *yaeT* outer-membrane assembly gene in “Pyropristinus,” and several other outer-membrane associated proteins (Bos et al., 2007; Sutcliffe, 2011) in both candidate phyla.

### Ecological distribution

**Figure 8:**
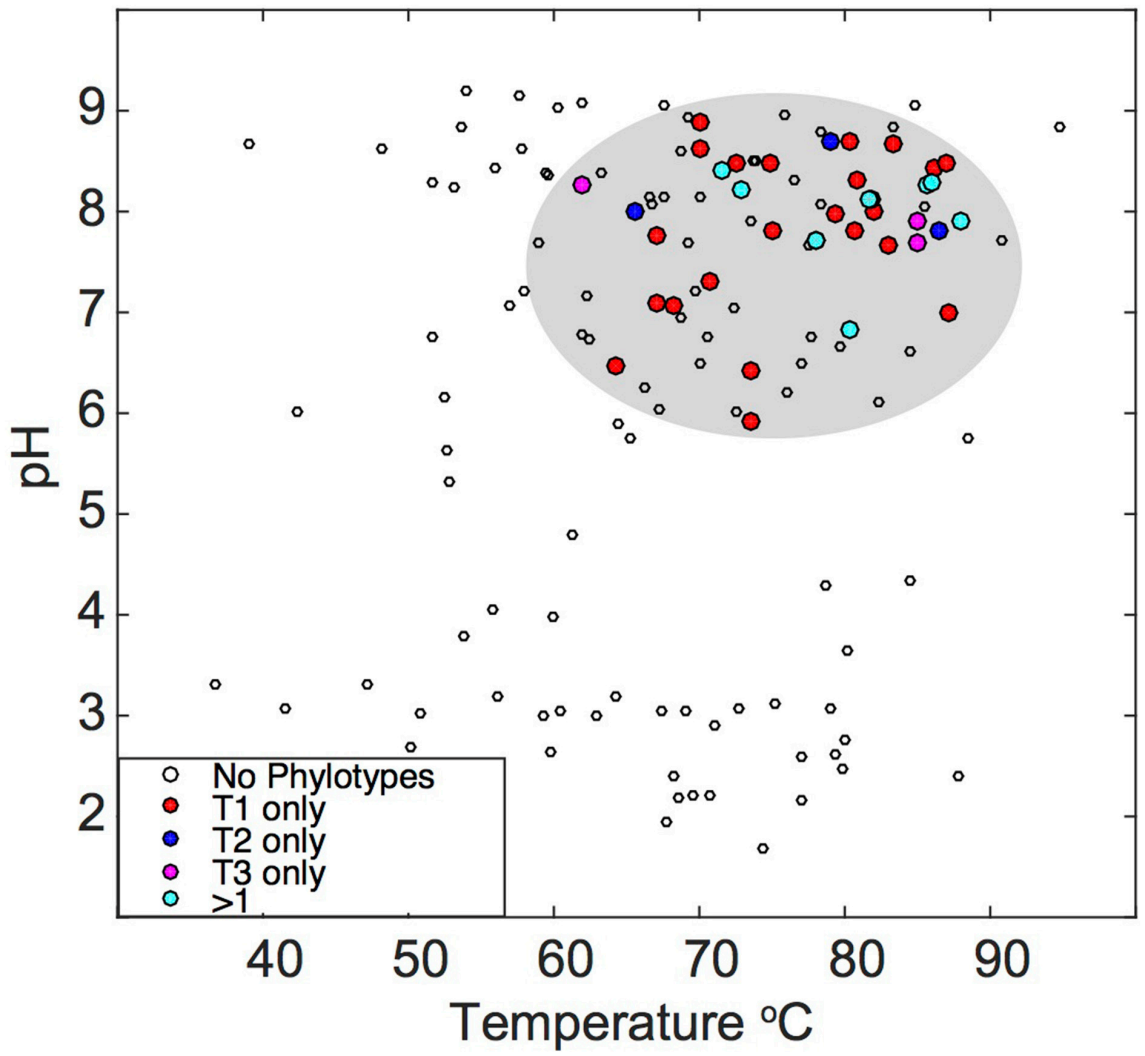
**Temperature and pH distribution of the “Pyropristinus” and Calescamantes phylotypes detected across different geothermal habitats**. Sequences (16S rRNA gene) sharing >97% nucleotide identity to the “Pyropristinus” and Calascamantes-OS population were identified from prior 16S rRNA gene surveys of YNP and publically available databases [red = “Pyropristinus” Type 1 only (*n* = 24); blue = “Pyropristinus” Type 2 only (*n* = 3); purple = Calescamantes-OS only (*n* = 3); cyan = 2-3 phylotypes present (*n* = 8); open circles = none of the three phyla detected]. Sites not containing these lineages are only shown for datasets that extensively surveyed YNP hot springs with universal bacterial PCR primers.

Previously compiled datasets of 16S rRNA gene diversity in YNP and public 16S rRNA gene databases were queried for the presence of “Pyropristinus” Types 1 and 2, and Calescamantes-OS populations. The presence of similar populations (>97% 16S rRNA gene identity) is currently restricted to terrestrial thermal springs, largely in affiliation with Aquificales “streamer” communities (Supplementary Table 2). No representatives were found in marine hydrothermal settings based on searches against public 16S rRNA gene databases. Moreover, these populations were only detected in high-temperature (pH ~6–9) geothermal springs, and only one sequence similar to “Pyropristinus” Type 2 has been observed outside of YNP (Figure 8; Supplementary Table 2). This analysis was restricted to matches exhibiting >97% nt identity to the three phylotypes and thus excludes more distantly related phylotypes that have been detected in other systems (for instance hydrothermal vents at the Southern Okinawa Trough and Great Boiling Spring, Nevada; Nunoura et al., 2010; Dodsworth et al., 2011). The temperature and pH range of sites used to infer phylotype distribution (Takacs-Vesbach, unpublished) was highly-similar statistically to the range observed for thermal springs within the entire YNP ecosystem (Pearson's *r* = 0.66, *P* < 0.05; Supplemental Figure 4), which indicates that this dataset was appropriate for inferring the presence or absence of these three populations with respect to temperature and pH within YNP. The observed temperature and pH ranges for “Pyropristinus” (T1 and T2) and the Calescamantes-OS phylotypes were not significantly different from one another (*P* ≥ 0.05), which suggests that they all occupy similar physicochemical niches. These results are also consistent with earlier observations of closely related EM3 and EM19-like populations in high-temperature, circumneutral communities dominated by *Thermocrinis* spp. (Aquificae) (Reysenbach et al., 1994; Blank et al., 2002; Meyer-Dombard et al., 2011).

## Conclusions

Results from phylogenetic analyses showed that two of the new phylotypes characterized here (Type 1 and Type 2) represent different groups of a phylum distinct from other characterized bacterial phyla, and for which we propose the candidate genera epithets “*Candidatus* Caldipriscus sp. T1” (Cal'di.pris.cus. L. masc. adj. *caldus*, hot; L. masc. n. *priscus*, ancient or primitive; ancient thermophile) and “*Candidatus* Thermoproauctor sp. T2” (Ther.mo.pro.auc'tor. Gr. fem. n. *therme*, heat; L. masc. n. *proauctor*, ancestor/founder; thermophilic ancestor), respectively. Further, on the basis of phylogenetic evidence, we propose the candidate phylum-level name “Pyropristinus” (Pyr.o'pris.tin.us. Gr. neutr. n. *pyr*, fire; L. masc. adj. *pristinus*, former/early; early thermophiles) to include the *Ca*. Caldipriscus, *Ca*. Thermoproauctor, and other closely related uncultured organisms, inclusive of the formerly identified EM3. The “Pyropristinus” and Calescamantes populations described here appear to use reduced sources of organic C to respire aerobically, and likely rely on C sources from other autotrophic members of the “streamer” communities. The consistency with which the “Pyropristinus” and Calescamantes lineages co-occur with *Thermocrinis* spp. (Aquificales) in streamer environments suggests that these early branching bacteria may have co-evolved in circumneutral high-temperature environments. Further, metabolic reconstruction suggests these organisms play an important role in C cycling of these high temperature ecosystems. Differences in energy conservation mechanisms between the “Pyropristinus” and Calescamantes lineages (e.g., ATPases, potential to respire anaerobically) suggests that they occupy different microenvironments across oxygen gradients. Importantly, these newly-described phylotypes provide increased resolution of the metabolic attributes associated with deep-branching thermophilic bacterial lineages.

## Author contributions

ZJ, WI, KM, CT, and DR either participated in sample acquisition, DNA extraction, and/or metagenome sequence analysis. DC, ZJ, WI, DR, RJ, and CT analyzed and interpreted the assembled sequence. DC, WI, ZJ, RJ, and CT contributed to manuscript preparation; DC, WI, and CT wrote and reviewed the manuscript.

### Conflict of interest statement

The authors declare that the research was conducted in the absence of any commercial or financial relationships that could be construed as a potential conflict of interest.
