# Supplementary material for: Novel, Deep-Branching Heterotrophic Bacterial Populations Recovered from Thermal Spring Metagenomes
Source: Front Microbiol. 2016 Mar 15;7:304. doi: 10.3389/fmicb.2016.00304 (PMC4791363; doi:10.3389/fmicb.2016.00304)
Supplement: Supplementary file 5 [file DataSheet1.DOCX]

**Legend for Supplementary Tables and Figures**

**Supplementary Table 1. Taxonomic information for references used in the phylogenomic analyses.**

**Supplementary Table 2. Metadata for samples from databases/datasets that were used to survey the distribution of the three lineages.**

**Supplementary Table 3. List of phylum-specific conserved signature indels (CSIs) for Thermotogae and Aquificae that were used as references for the T1-T3 lineages**

**Supplementary Table 4. List of pathways with presence/absence of genes used to infer the metabolic potential of the T1, T2 and T3 lineages.**

**Supplemental Figure 1. Coverage and G + C (%) analysis of contigs corresponding to separate assemblies of T1.1, T1.2, T2.1 and T3.1 populations.** Cumulative sequence is reported on the right y-axis (dashed line) on the left panels and individual scaffolds are plotted by G+C % (left y-axis). Right panels show G+C (%) as a function of scaffold coverage for (**A)** T1.1 (top) and T1.2 (bottom) and **(B)** T2.1 (top) and T3.1 (bottom)

**Supplemental Figure 2. Maximum likelihood phylogenetic tree of ribosomal proteins of the 'Pyropristinus' and Calescamantes lineages.** ML tree of a 5 (4 universal, 1 bacterial-only) ribosomal protein concatenation. Twenty-seven archaeal references were used as outgroups. Phyla with more than one reference are collapsed. Bootstrap values (100 replicates) are given at the nodes where >50%. Scale shows expected substitutions per site.

**Supplemental Figure 3. Maximum likelihood phylogenetic tree of the 'Pyropristinus' and Calescamantes lineages without outgroups.** ML tree of a concatenated single-copy housekeeping 18 gene dataset. Phyla with more than one reference are collapsed. Bootstrap values (100 replicates) are given at the nodes where >50%. Scale shows expected substitutions per site.

**Supplemental Figure 4. Sampling density maps for the YNP geothermal ecosystem and sites used to infer 'Pyropristinus' T1 and T2, and Calescamantes-OS distribution. (A)** Sampling density of springs used to infer distribution of 'Pyropristinus' T1, T2 and Calescamantes-OS by temperature and pH of springs (n=141). **(B)** Sampling density of springs in the YNP Research Coordination Network database (n=7680) by temperature and pH. Color scale indicates normalized sampling density for each dataset with dark blue indicating lowest density and dark red indicating highest density.
