# Supplementary figures and images for: Novel, Deep-Branching Heterotrophic Bacterial Populations Recovered from Thermal Spring Metagenomes
Source: Front Microbiol. 2016 Mar 15;7:304. doi: 10.3389/fmicb.2016.00304 (PMC4791363; doi:10.3389/fmicb.2016.00304)

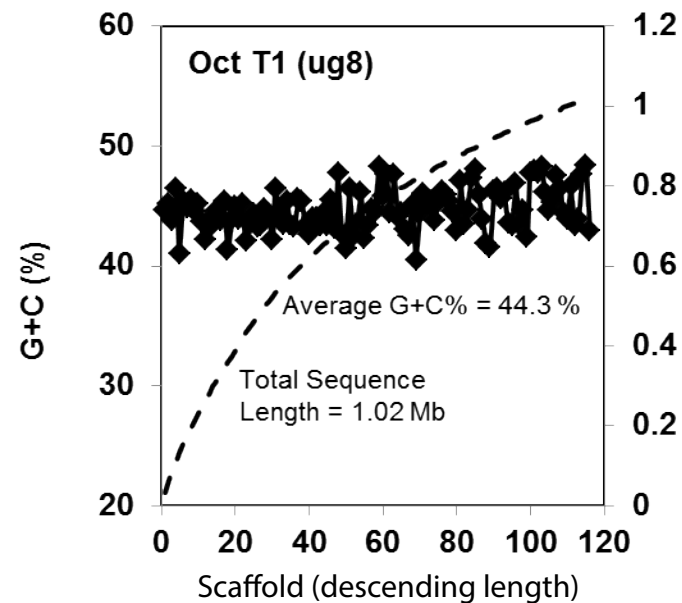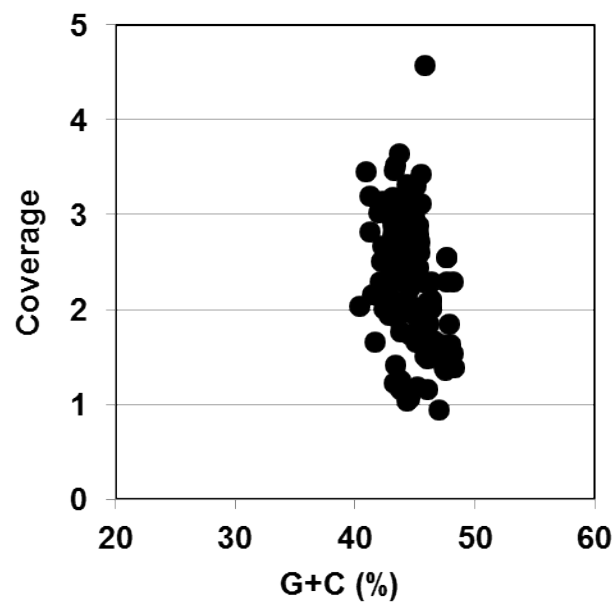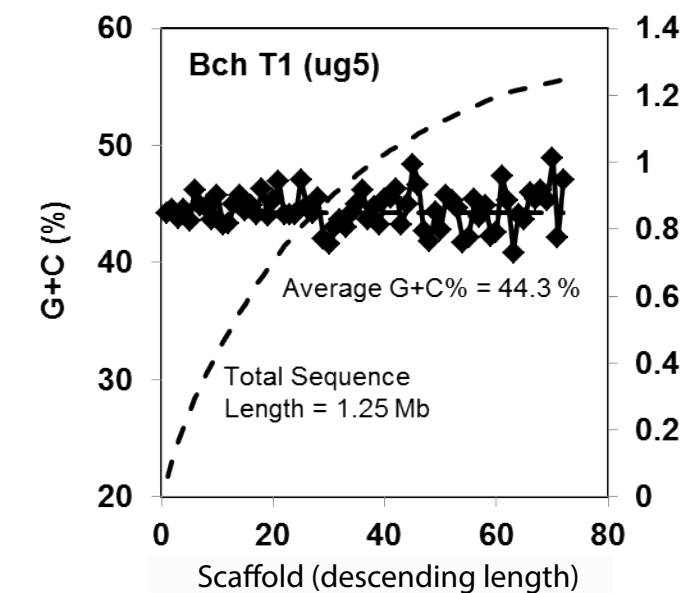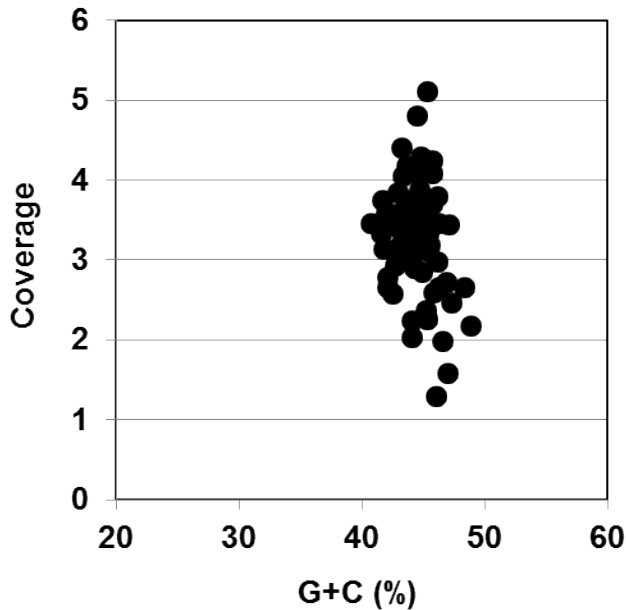

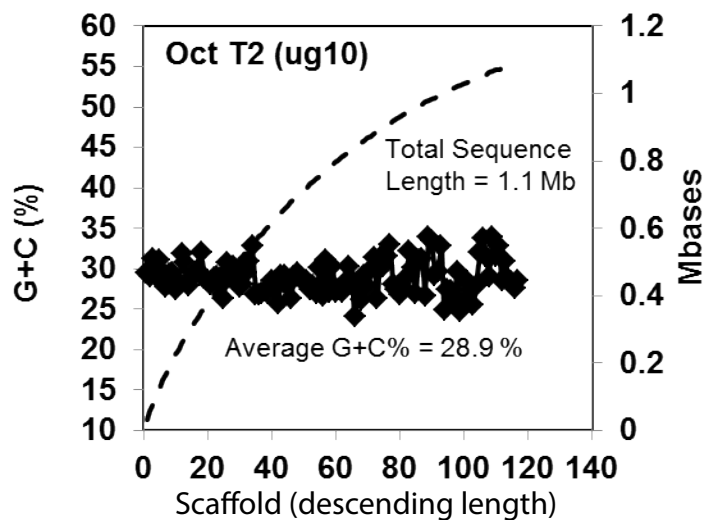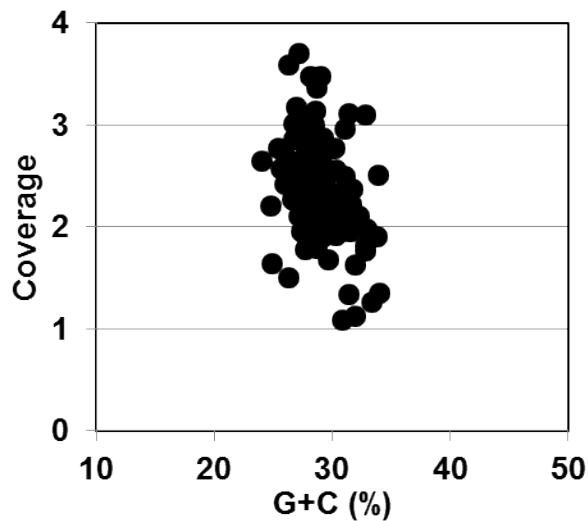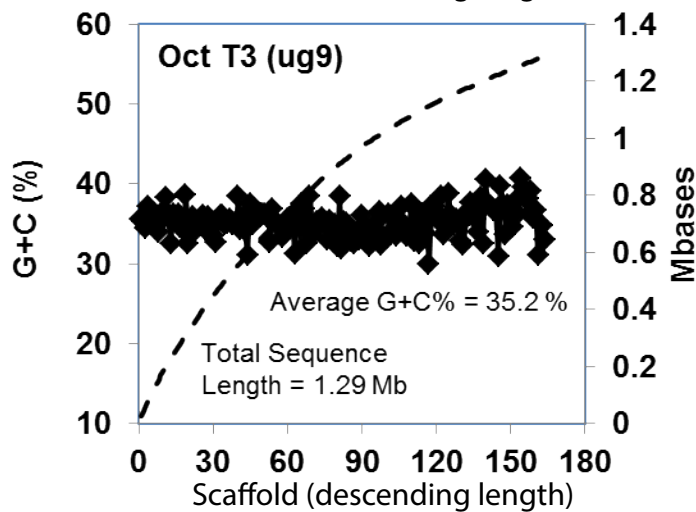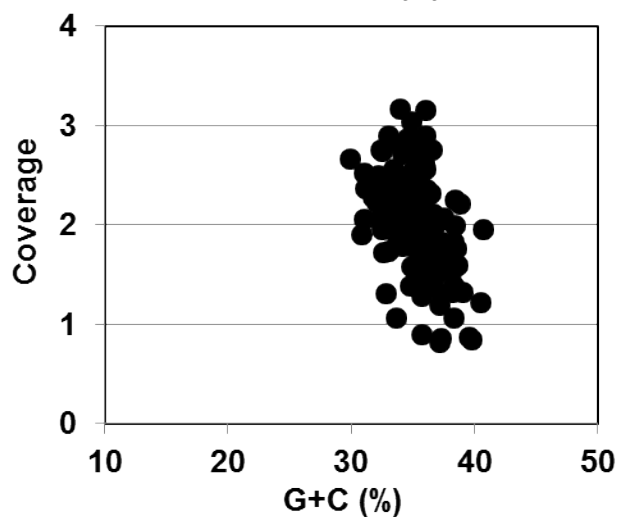

Supplement: Supplementary file 6 [file Image1.PDF]

Figure S2

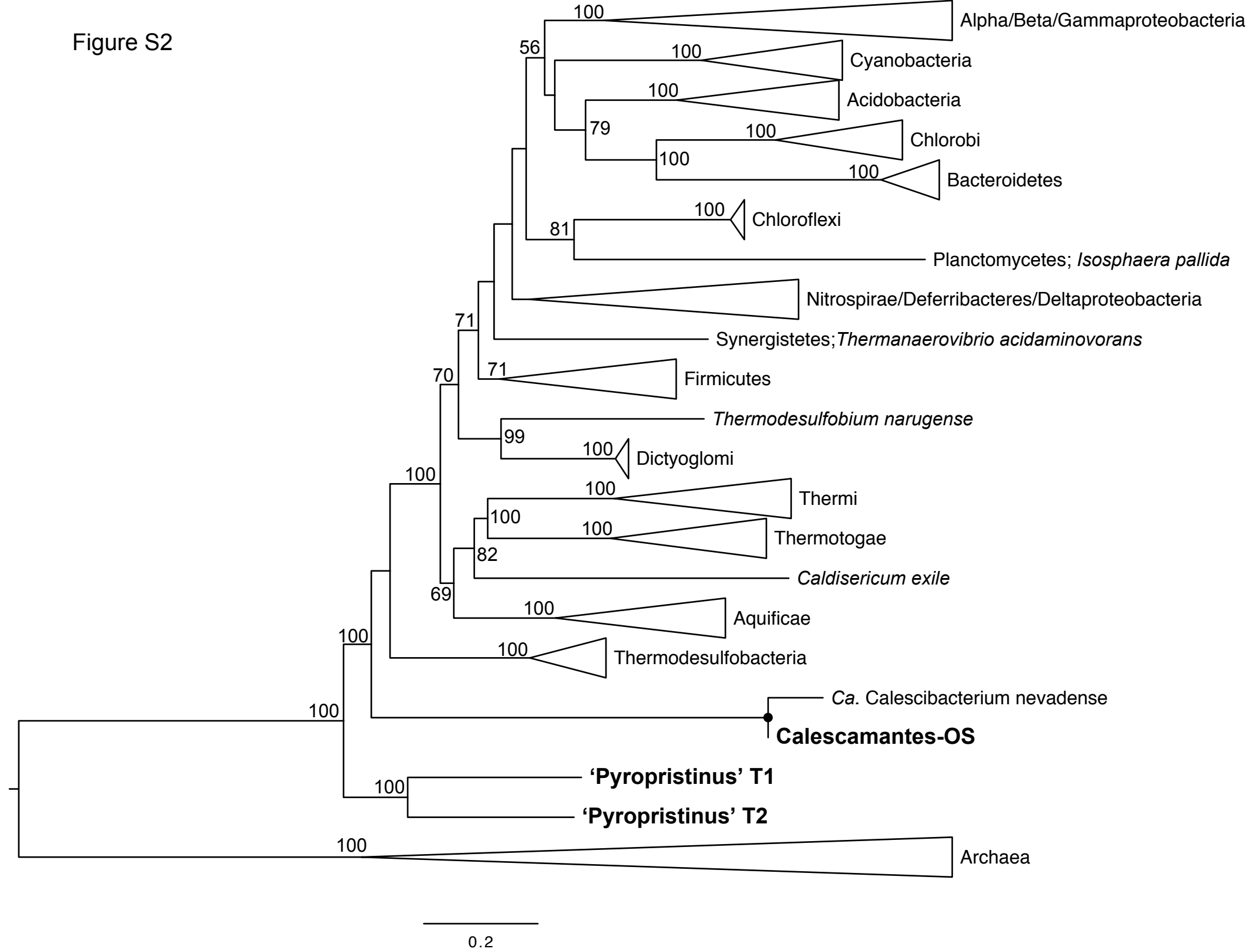

Supplement: Supplementary file 7 [file Image2.PDF]

Figure  
S3

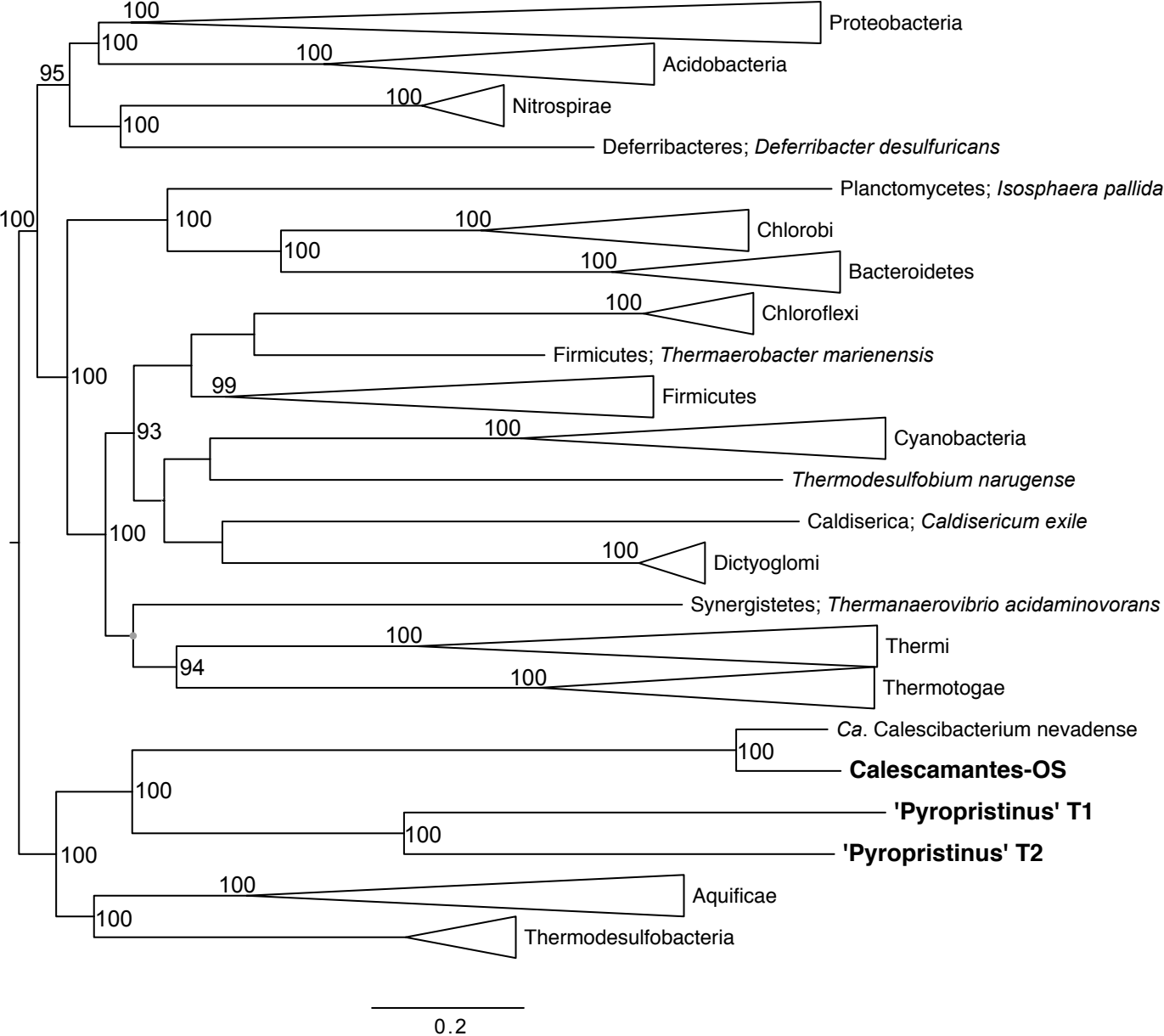

Supplement: Supplementary file 8 [file Image3.PDF]

Figure S4

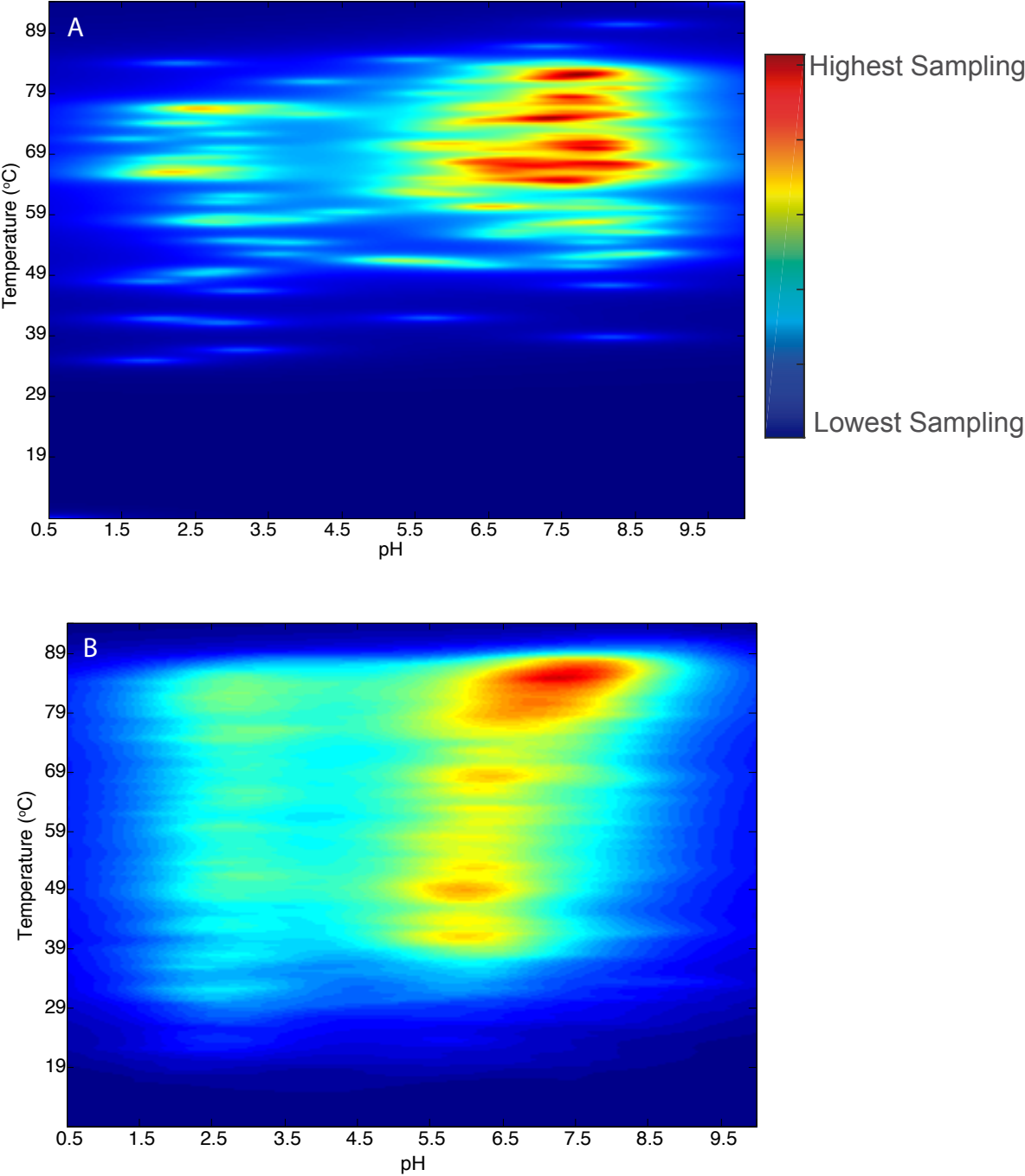

Supplement: Supplementary file 9 [file Image4.PDF]
